# A study on the effect of symbiotic fermented milk products on human gastrointestinal health: Double‐blind randomized controlled clinical trial

**DOI:** 10.1002/fsn3.2890

**Published:** 2022-05-03

**Authors:** Wenyan Liao, Miya Su, Dong Zhang

**Affiliations:** ^1^ State Key Laboratory of Dairy Biotechnology Shanghai Engineering Research Center of Dairy Biotechnology Dairy Research Institute Bright Dairy & Food Co., Ltd Shanghai China

**Keywords:** gastrointestinal health, Inulin, *L. plantarum* ST‐III, symbiotics

## Abstract

Several studies have claimed that the consumption of fermented dairy products can improve human gastrointestinal (GI) health. However, the numbers of systematic clinic trials are limited. In this study, a yogurt containing both probiotics and prebiotics was developed and a double‐blind randomized controlled clinical trial was carried out to evaluate the effect of the product on human gastrointestinal health in three different aspects: (1) the effect on functional constipation (FC) and functional diarrhea (FD); (2) the effect on gastrointestinal (GI) tract immune system; and (3) the changes in GI tract microbiota. Participants who suffered FC or FD were randomized into three groups (*n* = 66 each group): the first group was treated with fermented milk with *Lactobacillus plantarum* ST‐III (7 mg/kg) and inulin (1.5%), the second group was treated with *L*. *plantarum* ST‐III (7 mg/kg) and inulin (1.0%), and the third group (control group) was treated without probiotics and prebiotics. Half of the participants stopped the treatment after 14 days and the rest of the group continued the trial to the full 28 days. The fecal samples of participants were analyzed regarding their short‐chain fatty acids (SCFAs), secretory immunoglobulin A (sIgA), and microbiota. A survey on GI tract health was conducted and the Bristol stool scale was recorded. The results showed that the consumption of the symbiotic yogurt for 14 days and 28 days can both improve the digestive system, with the continual consumption of product containing *L*. *plantarum* ST‐III (7 mg/kg) and inulin (1.5%) for 28 days showing the most significance. The consumption of this product may be used as a potential functional food.

## Graphical abstract

The objectives of this study were to develop a symbiotic fermented food product containing both a prebiotic ingredient and probiotic cultures as well as analyzing the changes in gut microbiota after consumption. The effect of the symbiotic products on the human gastrointestinal health via clinical trials was evaluated.

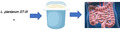

## 1 INTRODUCTION

Consumption of probiotics and prebiotics in the form of fermented dairy products has been widely used as functional foods for enhancing human gut health in the last few decades (Zepeda‐Hernández et al., 2021). The concept of symbiotics, which includes both probiotics and prebiotics within a food, has been gaining interest in recent times. The individual benefits of probiotics and prebiotics on human digestive health have been widely studied and results show that they have numerous positive effects on the human digestive system, such as improved balance of colonic microflora, bowel habits, and treatment of diarrhea (Mansour et al., 2021; Yan, 2020). However, the combined effect of probiotics and prebiotics taken together has not been studied widely. Probiotics contain live beneficial microbes that can improve digestive health. The probiotic *Lactobacillus plantarum* was reported to influence human gut health such as (Kusumo, Maulahela, et al., 2019, 2019,2019, 2019) improving the mucosal immune function and helping against pathogens such as *Clostridia* spp. It also helps in balancing the gut microbiota by producing short‐chain fatty acids as an energy source to maintain the gut's ecosystem and physiology. Dysbiosis is correlated with SCFAs imbalance which in turn results in functional constipation, whereas intake of *L*. *plantarum* can influence all the SCFAs parameters (acetate, propionate, and butyrate), which helps relieve the symptoms. *L*. *plantarum* ST‐III is a well‐known probiotic bacteria strain that is used in fermented dairy products (Yan et al., 2018). *L*. *plantarum* ST‐III was first isolated from kimchi based on its ability to temporarily persist in plants, the insect intestine, and in the intestinal tract of vertebrate animals (Zang et al., 2019; Zhao et al., 2014). It has an exceptional ability to bind to intestinal mucosa (the innermost layer of the gastrointestinal tract), which can increase the gut's population of beneficial bacteria (Liu et al., 2019). Several studies have been carried out to test the effectiveness of *L*. *plantarum* ST‐III, which can modulate the human gut microbiota and alleviate intestinal metabolic disorders, such as functional constipation (FC) and functional diarrhea (FD) (Zepeda‐Hernández et al., 2021; Zhao et al., 2014). Prebiotics are mainly carbohydrate‐based food ingredients that cannot be digested in the small intestine, but pass to the large intestine where they are utilized by probiotics to improve gut microflora (Rezende et al., 2021). Inulin is a common prebiotic compound that exists in the roots of many plants such as chicory. The health benefits of inulin have been extensively reviewed by Roberfroid in 2000 (Menne et al., 2000). The benefits include an increase in calcium and magnesium absorption, better control of blood sugar, and it can promote the growth of probiotic cultures (Arruda et al., 2020; Guaragni et al., 2020). Consumption of the symbiotic product may change human gut health, however, the clinical trials are limited and more clinical trials are needed (Zhang et al., 2019).

The objectives of this study were to develop a symbiotic fermented food product containing both a prebiotic ingredient and probiotic cultures and evaluate their effects on the human gut health after consumption. The effect of the symbiotic products on the human gastrointestinal health via clinical trials was evaluated.

## 2 MATERIALS AND METHODS

### 2.1 Preparation of symbiotic fermented milk products

Yoghurts with three different formulations (P2.0, P1.0, and control) were prepared according to the method of Li et al. (2016) with some slight modifications. Briefly, inulin powder was dissolved firstly in 60°C whole milk at a rate of 1.5% and 1.0% (w/w) using a mixer, for P2.0 and P1.0, respectively. The milk was then pasteurized in a water bath at 85ºC for 5 min. The milk was cooled down to 22°C using an ice‐water bath, and then inoculated with 100 U/kg starter culture F‐DVS BY‐Premium (*Lactobacillus acidophilus*, *Lactobacillus bulgaricus*, and *Streptococcus thermophiles*; Chr‐Hansen) and *L*. *plantarum* ST‐III (0.0007%, w/w). A product with only starter culture was also prepared as a control. The samples were fermented at 43°C for 5 h. The samples were stored at 4°C for further analysis.

### 2.2 Participants and study design

A total of 198 participants (ratio of male and female, 1:1) aged 25–45 years old with functional constipation or functional diarrhea were invited to this study. They also agreed not to take any drugs, supplements, or other dairy products during the trial. The participants were randomly assigned to three groups of 66 and each group was fed with P2.0, P1.0, or control products daily for 14 days. Half of the participants in each group were asked to stop taking the product while the rest continued up to 28 days. At the end of the trial, the data collected from 187 of the 198 participants were confirmed to be valid. The rest of the participants either dropped the test or the data collected from them were not valid. The amount of fermented milk consumed by participants daily was 250 g per day.

### 2.3 Evaluation of the participants’ gastrointestinal health

#### 2.3.1 The analyses of the stool samples

SCFAs, sIgA, and microbiological composition (*Bifidobacteria*, *Lactobacillus*, *Clostridium perfringens*, and *Escherichia coli*) of the stool samples were analyzed using GC‐MS, enzyme‐linked immunosorbent assay, and culture‐dependent methods, respectively. The stool samples were collected after the product consumption.

#### 2.3.2 Survey

A survey based on “intestinal health status,” “defecation habit satisfaction,” “digestive system improvement,” “hospital anxiety,” and “depression scale” was designed and collected at day 1, day 3, day 7, day 14, day 21, and day 28.

#### 2.3.3 Bristol stool scale

The Bristol stool scale is a diagnostic medical tool designed to classify the form of human feces into seven categories, as shown in Table 1. The shape of the patient's stool was recorded at day 1, day 3, day 7, day 14, day 21, and day 28.

**TABLE 1:** Bristol stool scale

Score	Description
1	Separate hard pellets, like nuts.
2	Sausage‐shaped but lumpy and hard to pass.
3	Sausage‐shaped but lumpy and easier to pass.
4	Like a sausage or snake, smooth and soft.
5	Soft blobs with clear‐cut edges.
6	Fluffy pieces with ragged edges, a mushy stool.
7	Watery, no solid pieces, entirely liquid.

### 2.4 Statistical analysis

The effect of consumption of the yogurt product on the patient's gut health was statistically analyzed and compared using a homogeneity of variance test and Bonferroni posttest by SPSS statistical software version 21 (SPP Inc.,). A 0.05 was considered as significant differences for all analyses.

## 3 RESULTS AND DISCUSSION

### 3.1 The percentage of volunteers completing the trial

A total of 472 people initially volunteered in this experiment. One hundred and ninety‐eight participants were eventually selected for the assay. They were randomly divided into three groups: P2.0, P1.0, and control products, with 66 people in each group. Eleven participants dropped off, including four in the P2.0 group, one in the P1.0 group, and six in the control group. The overall dropout rate was 5.6%. No replacement of volunteers occurred during the experiment. A total of 187 volunteers completed the trial and were included in the statistical analysis.

### 3.2 The analyses of the stool samples

Table 2 shows the changes in *Bifidobacteria, Lactobacillus, C*. *perfringens, and E. coli* after 14 days consumption of P2.0, P1.0, and the control. The results show that there were no significant differences in the microbiota of the stool samples at day 1, whereas there were significant changes after 14 days consumption of the products. Prebiotics are mostly fibers that are nondigestible food ingredients and beneficially affect the host's health by selectively stimulating the growth and/or activity of some genera of microorganisms in the colon, generally *Lactobacilli* and *Bifidobacterial*. *Bifidobacteria* can be isolated from the feces of humans and animals. In 1974, *Bifidobacteria* were first isolated from a healthy child and then it was named by modern taxonomic tools in 1990 (Kuczius, 2009). *Bifidobacteria* have been reported to be beneficial in digestive health. Because of its health benefits, it has been reported that more than 70 dairy products containing *Bifidobacteria* spp. can be found in the global functional food markets (Antunes, et al., 2009). Many research studies have shown that the consumption of *Bifidobacteria* has significant effects not only on traveler's diarrhea, amitotic‐associated diarrhea, and childhood diarrhea, but also prevention of cancer, reduction in blood cholesterol levels, and relief of lactose intolerance symptoms (Tahri, et al,.^1995^). *Lactobacillus* spp. are important members of the human gut microbiome. They help prevent disease, and they also help prevent pathogens from colonizing the gut, including the release of antimicrobial substances in response to invaders. *Lactobacillus* breaks down dietary fibers and phytonutrients (like polyphenols) which have beneficial effects on human health. These microbes produce several important substances, including lactic acid, SCFAs, and some antimicrobial substances to deter opportunistic bacteria from disrupting the gut ecosystem and human health. The production of lactate and acetate from carbohydrates (sources of prebiotic dietary fibers) is an important factor in the pH of the gut; these help to keep the acidity of the gut balanced in a way that encourages beneficial and commensal (harmless) species while deterring invaders that could cause sickness. The butyrate produced by gut bacteria is essential for a healthy GI tract, especially as it provides 70% of the energy used by the intestinal epithelial cells. Therefore, not only do these probiotic bacteria help to keep the gut healthy, they enable other bacterial species in the gut to thrive as well. *C*. *perfringens* can be found on raw meat and poultry, in the intestines of animals, and in the environment. If ingested, these bacteria can produce a toxin (poison) that causes diarrhea. Outbreaks tend to happen in places that serve food to large groups of people, such as hospitals, school cafeterias, prisons, and nursing homes, and at events with catered food. *C*. *perfringens* outbreaks occur most often in November and December in Northern hemisphere. Many of these outbreaks have been linked to foods commonly served during the holidays, such as turkey and roast beef. Centre of Disease Control (CDC) accepts specimens only from foodborne outbreaks for testing. Oral rehydration can be used to prevent or treat dehydration, or in severe cases, intravenous fluids with electrolytes can be used. Antibiotic treatment is not recommended for treating *C*. *perfringens*. *E*. *coli* normally lives in the intestine. Most strains are usually harmless. A few strains cause diarrhea/bloody diarrhea, vomiting, stomach pains, and cramps. One strain can lead to kidney failure if not properly managed. Eating contaminated food is the most common way to get an *E*. *coli* infection. Most people recover within a week without medications. In summary, the results indicate that consumption of the symbiotic yogurt can increase the population of beneficial bacteria (*Bifidobacteria* and *Lactobacillus*) and reduce the population of harmful bacteria like *C*. *perfringens* and *E*. *coli*. This indicates that the product can improve gut health and prevent constipation.

**TABLE 2:** The changes of microbiota after 14‐day consumption of P2.0, P1.0, and the control

Date	Bacteria	P2.0	P1.0	Control	*p*‐ value
Day 1	*Bifidobacteria*	5.03 ± 0.92	5.1 ± 1.11	5.01 ± 0.97	.932
	*Lactobacillus*	4.96 ± 0.92	5.11 ± 1.47	5.04 ± 1.26	.888
	*Clostridium perfringens*	5.58 ± 1.56	5.45 ± 1.36	5.65 ± 1.64	.867
	*Escherichia coli*	5.42 ± 1.54	5.56 ± 1.93	5.49 ± 1.34	.945
Day 14	*Bifidobacteria*	6.17 ± 1.04	6.00 ± 1.16	5.17 ± 1.01	.001
	*Lactobacillus*	6.53 ± 1.01	5.95 ± 0.87	4.97 ± 0.88	.001
	*Clostridium perfringens*	4,64 ± 1.30	4.74 ± 1.57	5.63 ± 1.21	.010
	*Escherichia coli*	4.59 ± 1.14	4.74 ± 1.14	5.54 ± 1.47	.008

Table 3 shows the summary of fecal SCFAs and sIgA contents of volunteers from each group with functional constipation during the trial. There was no significant difference in fecal SCFAs and sIgA contents among all groups at baseline. After 14 days consumption of the products, there were significant differences in fecal acetic acid, and total SCFAs and sIgA contents among the three groups.

**TABLE 3:** The changes of short‐chain fatty acids (SCFAs, ppm) and sIgA (IgA) after 14‐day consumption of P2.0, P1.0, and the control

Date	Items	P2.0	P1.0	Control	*p*‐value
Day 0	Acetic acid	0.358 ± 0.094	0.356 ± 0.090	0.361 ± 0.100	.978
	Propionic acid	0.139 ± 0.068	0.135 ± 0.050	0.136 ± 0.047	.963
	Butyric acid	0.093 ± 0.049	0.091 ± 0.048	0.095 ± 0.035	.951
	Total SCFAs	0.592 ± 0.112	0.583 ± 0.120	0.589 ± 0.156	.959
	sIgA	405.10 ± 201.34	404.46 ± 200.46	403.15 ± 159.64	.999
Day 14	Acetic acid	0.457 ± 0.115	0.452 ± 0.097	0.356 ± 0.122	.001
	Propionic acid	0.150 ± 0.057	0.147 ± 0.051	0.137 ± 0.038	.557
	Butyric acid	0.098 ± 0.032	0.094 ± 0.045	0.093 ± 0.049	.904
	Total SCFAs	0.705 ± 0.124	0.693 ± 0.132	0.586 ± 0.148	.001
	sIgA	530.24 ± 236.68	521.31 ± 194.83	402.39 ± 131.43	.018

There is now an abundance of evidence to show that short‐chain fatty acids (SCFAs) play an important role in the maintenance of health and the development of disease. SCFAs are a subset of fatty acids that are produced by the gut microbiota during the fermentation of partial and nondigestible polysaccharides. SCFAs are by‐products of healthy gut microbes after they interact with dietary fiber. To be specific, SCFAs support immunity, mucosal health, leaky gut, inflammation, and probiotic benefits. The main SCFAs produced in the gut are butyrate, propionate, and acetate. They are predominantly produced in the colon. SCFAs can be found in other areas of the intestines and can circulate in the bloodstream to help support the liver, lipid balance, cell growth, energy production, etc. (Mirzaei et al., 2021). Butyric acid is the most frequently studied of the SCFAs and is also a primary fuel source for colonocytes (Qaisrani et al., 2015). Functionally, SCFAs may also support blood flow to the colon, mineral absorption, healthy intestinal pH, normal bile and cholesterol metabolism, and the motility of material passing through. By promoting healthy intestinal pH, SCFAs provide antimicrobial and probiotic benefits as pH can dictate what types of bacteria are able to grow (Pratiwi Dyah Kusumo, Maulahela, et al., 2019, 2019,2019, 2019; LaGamma et al., 2021; Nishida et al., 2021).

sIgA from saliva tends to make its way into the gut. The amount of sIgA produced largely depends on the presence of antigens in the gut. The more antigens that are present, then the higher its production. sIgA identifies two types of antigens. The first type are enteric pathogens that secrete toxins and cause infections. The second type is commensal microflora or beneficial bacteria, for example, probiotics. Studies show that sIgA can help get rid of harmful microbes and reshape the gut ecology by increasing the beneficial microbes (Kusumo, Maulahela, et al., 2019, 2019,2019, 2019). It is possible to deal with low or high sIgA levels through intake of the beneficial microbes via symbiotic products. Many health issues start in the gastrointestinal tract and the body's sIgA production also tends to decline with age. Compromised gut health due to stress then activates the immune system. This leads to the formation of antibodies like sIgA. This allows the immune system to fight threats to physical health. However, there can be imbalances in the sIgA level arising from fluctuations in cortisol production (Strazdins et al., 2005). This may give rise to illnesses like irritable bowel syndrome (IBS) (Xue et al., 2020). In this study, the enhancement of SCFAs and sIgA can promote the balance of participants’ gut microbiota and the GI tract immune system.

### 3.3 Survey

#### 3.3.1 Participants with functional constipation (FC)

The health status of the participants with FC and FD was evaluated based on the data relating to defecation time, frequency, status, and the number of unsmooth defecations based on Bristol scale per day. Table 4 shows the scores of intestinal health for each group of participants with functional constipation during the trial (lower scores indicate better intestinal health). During the time when all volunteers were taking the product, from day 3 of intervention, there were significant intergroup differences in intestinal health status score by total score (the defecation time, frequency, status, and the number of unsmooth defecations per day). Among volunteers who continued to take the product after 14 days, the results showed significant differences between each item on days 21 and 28. Among volunteers who discontinued the product after 14 days, significant intergroup differences persisted only until day 21 with no significant intergroup differences in all scores at day 28.

**TABLE 4:** The total score of the participants with functional constipation (FC)

Date	P2.0	P1.0	Control	*p*‐value
Day 0	8.19 ± 1.40	8.33 ± 1.24	8.30 ± 1.80	.927
Day 3	6.26 ± 1.63	7.82 ± 1.24	8.53 ± 1.20	.001
Day 7	5.68 ± 1.47	7.39 ± 1.27	8.27 ± 1.46	.001
Day 14	4.39 ± 1.45	5.00 ± 1.46	8.20 ± 1.27	.001
Day 21	3.00 ± 1.54	3.25 ± 1.81	8.07 ± 1.44	.001
Day 28	2.35 ± 1.41	2.44 ± 1.55	7.39 ± 1.49	.001

In comparison between the volunteers with FC, the total score of P2.0 group tested in 3 and 7 days were significantly lower than the P1.0 group, suggesting the intestinal health is significantly better than the group taking ordinary yogurt volunteers. There was no significant difference in intestinal score between the volunteers who took P1.0 product and the ordinary yogurt group on the 3rd day of the experiment, however, the score of the frequency of defecation and the total score of intestinal health status on the 7th day were significantly higher than that of the ordinary yogurt group. At the 14th day of the experiment, the intestinal scores of the volunteers in the 1.0 and 2.0 product groups were significantly lower than those in the ordinary yogurt group, while there was no significant difference in the intestinal scores between the two product groups.

On days 21 and 28 of the trial, the intestinal scores of volunteers with functional constipation in the P1.0 and P2.0 groups who had taken the product for 28 days were still significantly lower than those in the normal yogurt group, and there were no significant differences between the two product groups. In the volunteers who stopped using the product, the scores of the P2.0 group were still significantly lower than those of the ordinary yoghurt group on the 21st day, and the total scores of the P1.0 group were also significantly lower than those of the ordinary yoghurt group. There were no significant differences in the scores of the two experimental product groups. At day 28, there were no significant differences in scores among the three groups of volunteers who had stopped using the product. Comparison of intragroup differences from baseline showed that all intestinal health scores and total scores of volunteers with functional constipation were significantly reduced from day 3 after taking the P2.0. The scores of the volunteers who continued to take the product after 14 days were also significantly lower than the baseline scores. The scores of the volunteers who stopped using the product were still significantly lower than the baseline scores on day 21, except for the defecation status score. However, there was no significant difference in the intestinal scores on day 28 compared to the baseline. The scores of defecation frequency and intestinal health status of functional constipation volunteers who received product 1.0 were significantly lower than those of baseline from day 7, while the scores of other intestinal conditions were significantly lower than those of baseline from day 14. Those who continued taking the product had significantly lower intestinal scores than baseline on days 21 and 28, while those who discontinued the product remained significantly lower than baseline on day 21, but did not differ significantly from baseline on day 28. There was no significant change in the intestinal health scores of the functionally constipated volunteers who received plain yogurt during the trial.

#### 3.3.2 Participants with functional diarrhea (FD)

Table 5 showed the volunteers with FD during defecation satisfaction rating of each group of volunteers summary (a lower score indicates a higher degree of satisfaction) during all the volunteers taking products, excrement characters bowel habit satisfaction ratings, and defecation satisfaction scores from the 7th day of intervention on significant differences between groups, stool frequency satisfaction score was observed at day 14 significant differences between groups. For volunteers who continued to take the product after 14 days, the differences in the above four indicators were still significant on days 21 and 28. For volunteers who stopped using the product after 14 days, there was no significant difference on the satisfaction score of defeces between groups on days 21 and 28. Between the two groups compared (Table 5), found that volunteers for functional diarrhea, 7 days in the experiment, volunteers taking 2.0 products excrement characters, bowel habit score, and defecation satisfaction total score was significantly lower than (that satisfaction is significantly higher than) volunteers taking regular yogurt, and fecal character satisfaction score was significantly lower than taking 1.0 products of volunteers. There was no significant difference in the satisfaction score between the volunteers who took the control sample and product 1.0. On day 14 of the experiment, the fecal traits, defecation habits score, and defecation satisfaction score of volunteers in 1.0 and 2.0 product groups were significantly lower than those in the normal yogurt group, and the satisfaction score of defecation times in 2.0 group was also significantly lower than that in the normal yogurt group, but there was no significant difference in each score between 1.0 and 2.0 product groups. Among volunteers with functional diarrhea who continued to take the product past day 14, the significant difference at day 14 persisted through days 21 and 28 of the trial, and the 1.0 product group also had a significantly lower intestinal health status score for defecation times at day 28 than the normal yogurt group. However, there was no significant difference between the satisfaction scores of the other volunteers on days 21 and 28 after they stopped using the products. Between two groups compared (Table 5) found that 7 days into the experiment volunteers with functional diarrhea taking 2.0 products had results where excrement characters, bowel habit score and defecation satisfaction total score was significantly lower than (that satisfaction is significantly higher than) volunteers taking regular yogurt; and fecal character satisfaction score was significantly lower than volunteers taking 1.0 products. There was no significant difference in the satisfaction score between the volunteers who took normal yogurt and product 1.0. On day 14 of the experiment, the fecal traits, defecation habits score, and defecation satisfaction score of volunteers in 1.0 and 2.0 product groups were significantly lower than those in the normal yogurt group, and the satisfaction score of defecation times in 2.0 group was also significantly lower than that in the normal yogurt group, but there was no significant difference in each score between 1.0 and 2.0 product groups.

**TABLE 5:** The total score of the participants with functional diarrhea (FD)

Date	P2.0	P1.0	Control	*p*‐value
Day 0	6.13 ± 2.62	6.06 ± 2.37	5.8 ± 2.86	.874
Day 3	5.48 ± 1.77	5.75 ± 1.44	5.73 ± 1.48	.756
Day 7	4.06 ± 1.57	4.41 ± 2.18	5.70 ± 1.49	.007
Day 14	4.06 ± 1.57	4.41 ± 2.18	5.70 ± 1.49	.001
Day 21	3.67 ± 1.63	3.81 ± 2.07	6.38 ± 1.36	.001
Day 28	3.40 ± 1.80	3.50 ± 1.90	6.00 ± 1.75	.001

Among volunteers with functional diarrhea who continued to take the product after day 14, the significant difference at day 14 persisted through days 21 and 28 of the trial, and the 1.0 product group also had a significantly lower satisfaction score for defecation times at day 28 than the normal yogurt group. However, there was no significant difference between the satisfaction scores of the other volunteers on days 21 and 28 after they stopped using the products.

### 3.4 Bristol stool scale

#### 3.4.1 Bristol stool scale of participants with functional constipation (FC)

Table 6 shows the summary of the Bristol score for each group of volunteers with functional constipation during the trial. At baseline, the fecal traits of volunteers in each group were dry and hard (mean score was around 2) and dark (mean score was between 5 and 6). Significant intergroup differences in fecal traits and fecal color scores were observed for volunteers taking the product from day 3 of the intervention. Those who continued to take the product after 14 days showed persistent differences in fecal traits and color ratings, and remained significant on days 21 and 28. Among volunteers who discontinued the product after 14 days, significant intergroup differences persisted only until day 21, and there was no significant intergroup difference in Bristol scores at day 28. Pair comparison between groups (Table 6) showed that from day 3 of the experiment, the fecal traits score of volunteers with functional constipation who took 2.0 product was significantly higher than that of the ordinary yogurt group (indicating that the fecal traits were wetter and softer), and the fecal color score was significantly lower than that of the ordinary yogurt group (indicating that the fecal color was lighter). On day 7, the fecal traits score of the product 2.0 group was significantly higher than that of product 1.0 group, and fecal color score was significantly lower than that of product 1.0 group. At the 14th day, the fecal traits score of the volunteers receiving 1.0 product was significantly higher than that of the normal yogurt group, and the fecal color score was significantly lower than that of the normal yogurt group, while the two scores were not different from those of the 2.0 product group.

**TABLE 6:** The Bristol stool scale of the participants with functional constipation (FC)

Date	Item	P2.0	P1.0	Control	*p*‐value
Day 0	Shape	2.2 ± 0.63	2.24 ± 0.79	2.17 ± 0.59	.855
	Color	5.25 ± 1.15	5.39 ± 1.06	5.67 ± 1.21	.639
Day 3	Shape	2.71 ± 0.0.69	2.33 ± 0.78	2.20 ± 0.66	.018
	Colour	4.87 ± 0.85	5.27 ± 1.10	5.53 ± 0.94	.031
Day 7	Shape	3.03 ± 0.84	2.55 ± 0.67	2.13 ± 0.68	.001
	Colour	4.39 ± 0.67	5.00 ± 0.90	5.40 ± 1.00	.001
Day 14	Shape	3.03 ± 0.60	2.91 ± 0.72	2.17 ± 0.70	.001
	Colour	4.16 ± 0.58	4.33 ± 0.65	5.33 ± 0.99	.001
Day 21	Shape	3.41 ± 0.51	3.38 ± 0.50	2.33 ± 0.82	.001
	Colour	4.06 ± 0.43	4.13 ± 0.34	5.27 ± 1.22	.001
Day 28	Shape	3.41 ± 0.51	3.38 ± 0.50	2.47 ± 0.83	.001
	Colour	4.00 ± 0.35	4.06 ± 0.25	5.40 ± 1.12	.001

On the 21st and 28th days of the experiment, the fecal traits scores of volunteers with functional constipation in the 1.0 and 2.0 product groups who continued to take the product past day 14 were significantly higher than those in the ordinary yogurt group, and the fecal color scores were significantly lower than those in the ordinary yogurt group. There was no significant difference between the two test product groups. In the volunteers who stopped using the product at day 14, similar pair‐to‐pair differences only lasted until day 21, and there were no significant differences in fecal traits and color scores among the volunteers who stopped using the product in the three groups on day 28. Compared with the baseline group (Table 6), volunteers from taking 2.0 products 3 days, has a marked increase in wet soft (feces), excrement and urine color score than baseline significantly reduced (stool color becomes shallow), on the 14th day excrement characters mean increased to 3 min or so, excrement and urine color near average gradually reduced to four points. Those who continued to take the product after 14 days still had significant differences in fecal traits and color scores from baseline at 21 and 28 days. The fecal traits and color scores of the volunteers who discontinued the product also remained significantly different from baseline at day 21, while neither score was significantly different from baseline at day 28.

The volunteers with functional constipation who received product 1.0 had a significantly higher fecal trait score and a significantly lower fecal color score on day 14 compared to baseline. Volunteers who continued to take the product past day 14 still showed significant differences in both scores from baseline at days 21 and 28. For the volunteers who stopped using the product on day 14, the fecal trait score was significantly higher than the baseline score on day 21 but the fecal trait score on day 28 and fecal color score were not significantly different from the baseline on days 21 and 28. There were no significant changes in fecal traits and color scores among volunteers with functional constipation who received plain yogurt during the trial.

#### 3.4.2 Bristol stool scale of participants with functional diarrhea (FD)

Table 7 is a summary of the Bristol scores of each group of volunteers with functional diarrhea during the trial. At baseline, the stool of each group was soft (mean score between 5 and 6) and moderately light (mean score between 3 and4). When all volunteers took the product, the fecal character score showed significant intergroup difference from day 7 of intervention, and the fecal color score showed significant intergroup difference from day 14 of intervention. Differences in fecal traits and color ratings persisted among volunteers who continued to take the product after 14 days, and remained significant at days 21 and 28. Among volunteers who discontinued the product after 14 days, significant intergroup differences persisted until day 21 only in fecal trait scores, which had no significant intergroup differences at day 28, and in fecal color scores at days 21 and 28. Pair comparison between groups (Table 7) showed that at day 7 volunteers with functional diarrhea taking the 2.0 product had a significantly lower fecal character score than that of the normal yogurt group (indicating that the stool was more formed). At day 14, fecal trait scores in the 1.0 and 2.0 product groups were significantly lower than those in the plain yogurt group, and fecal color scores in the 2.0 product group were significantly higher than those in the plain yogurt group (indicating darker fecal color). There were no significant differences in fecal traits and color scores between 1.0 and 2.0 product groups during the trial period. After 14 days, the functional diarrhea volunteers who continued to take 2.0 product had significantly lower fecal traits scores and higher fecal color scores on days 21 and 28 than the normal yogurt group. For those who continued to take product 1.0 and their fecal character scores were significantly lower than those in the normal yogurt group at days 21 and 28, and their fecal color scores remained higher only at day 21 than those in the control yogurt group. In the discontinued volunteers, there were no significant pair differences between groups in fecal traits or color scores at days 21 and 28. Compared with the baseline group differences, volunteers with FD taking 2.0 product 7 days, their the excrement characters had significantly lower scores than the baseline more molding (feces), excrement, and urine color score significantly increased than the baseline (dark) of excrement and urine, feces traits in 14 days average gradually decreased to 4.5 or so. The mean color of feces gradually increased to nearly 4 points. Volunteers who continued to take the product after 14 days still had significant differences in fecal traits and color scores from baseline at days 21 and 28. Only the fecal trait score remained significantly lower than the baseline score at day 21, and no significant differences were found at day 28 or fecal color score at days 21 and 28. For functional diarrhea volunteers taking the 1.0 product, fecal trait scores were significantly lower at day 14 than at baseline, with an average of 4.7 points. Volunteers who continued to take the product after day 14 still had significant differences in fecal trait scores from baseline to days 21 and 28. The significant difference in fecal trait scores between the day 14 stop volunteers and baseline lasted only until day 21. The fecal color score was significantly higher than the baseline score only at day 21, with an average score of 3.6. The fecal traits and color scores of functional diarrhea volunteers who took plain yogurt did not change significantly during the trial period.

**TABLE 7:** The Bristol stool scale of the participants with functional diarrhea (FD)

Date	Item	P2.0	P1.0	Control	*p*‐value
Day 0	Shape	5.61 ± 0.56	5.59 ± 0.61	5.70 ± 0.60	.755
	Colour	3.35 ± 0.71	3.53 ± 0.72	3.35 ± 1.04	.673
Day 3	Shape	5.39 ± 0.72	5.44 ± 0.80	5.60 ± 0.62	.485
	Colour	3.55 ± 0.57	3.47 ± 0.62	3.43 ± 0.68	.761
Day 7	Shape	4.90 ± 0.83	5.28 ± 0.63	5.60 ± 0.50	.001
	Colour	3.74 ± 0.51	3.63 ± 0.61	3.57 ± 0.57	.469
Day 14	Shape	4.55 ± 0.77	4.72 ± 0.85	5.53 ± 0.57	.001
	Colour	3.87 ± 0.05	3.81 ± 0.64	3.41 ± 0.73	.016
Day 21	Shape	4.13 ± 0.64	4.31 ± 0.48	5.63 ± 0.50	.001
	Colour	3.93 ± 0.26	3.88 ± 0.34	3.31 ± 0.70	.001
Day 28	Shape	4.13 ± 0.64	4.25 ± 0.58	5.56 ± 0.63	.001
	Colour	4.00 ± 0.53	3.75 ± 0.58	3.31 ± 0.79	.017

## 4 CONCLUSIONS

In comparison with the control product, both P2.0 and P1.0 showed significant effect on changing the intestinal health status of the participants with FC and FD after day 3 and day 7, respectively. The effect of the P2.0 on intestinal health was faster than that of the P1.0, whereas both products achieved similar results after 14 days of continuous consumption. The numbers of *Bifidobacteria* and *Lactobacillus* in feces of the participants significantly increased after 14‐day consumption of P2.0 and P1.0, with the most activity effect from P2.0. The numbers of *C*. *perfringens* and *E*. *coli* were significantly reduced after 14 days consumption of both P2.0 and P1.0. The results indicate that both products can balance and regulate the ecology of the participants’ gut microbiota. The levels of fecal acetic acid and total SCFAs and SIgA in the participants with functional constipation and functional diarrhea were significantly higher than the baseline levels after 14 days consumption of both P2.0 and P1.0. It shows that the products had significant effects on improving intestinal immune function in the participants with FC and FD. In conclusion, the symbiotic fermented product containing *L*. *plantarum* ST‐III and inulin can improve the human gastrointestinal health in terms of functional constipation, functional diarrhea, the GI tract immune system, and the GI tract microbiota, and can potentially be used as a functional food for gut heath.

## AUTHOR CONTRIBUTION

**Wenyan Liao:** Conceptualization (equal); Data curation (equal); Formal analysis (equal); Funding acquisition (equal); Investigation (equal); Methodology (equal); Project administration (equal); Resources (equal); Software (equal); Supervision (equal); Validation (equal); Visualization (equal); Writing – original draft (equal); Writing – review & editing (equal). **Miya Su:** Conceptualization (equal); Data curation (equal); Formal analysis (equal); Funding acquisition (equal); Investigation (equal); Methodology (equal); Project administration (equal); Resources (equal); Software (equal); Supervision (equal); Validation (equal); Visualization (equal); Writing – original draft (equal); Writing – review & editing (equal). **Dong Zhang:** Writing – original draft (equal); Writing – review & editing (equal).

## CONFLICTS OF INTEREST

There is no conflict of interest associated with this manuscript.

## Data Availability

The data that support the findings of this study are available on request from the corresponding author. The data are not publicly available due to privacy or ethical restrictions.
